# One Step Synthesis of NiO Nanoparticles via Solid-State Thermal Decomposition at Low-Temperature of Novel *Aqua*(2,9-dimethyl-1,10-phenanthroline)NiCl_2_ Complex

**DOI:** 10.3390/ijms141223941

**Published:** 2013-12-09

**Authors:** Assem Barakat, Mousa Al-Noaimi, Mohammed Suleiman, Abdullah S. Aldwayyan, Belkheir Hammouti, Taibi Ben Hadda, Salim F. Haddad, Ahmed Boshaala, Ismail Warad

**Affiliations:** 1 Department of Chemistry, King Saud University, P.O. Box 2455, Riyadh 11451, Saudi Arabia; E-Mail: ambarakat@ksu.edu.sa; 2 Department of Chemistry, Hashemite University, Zarqa 13115, Jordan; E-Mail: manoaimi@hu.edu.jo; 3 Department of Chemistry, Science College, AN-Najah National University, P.O. Box 7, Nablus, Palestine; E-Mail: suleimanshtaya@najah.edu; 4 Department of Physics and Astronomy, King Saud University, P.O. Box 2455, Riyadh 11451, Saudi Arabia; E-Mail: dwayyan@ksu.edu.sa; 5 Laboratoire de Chimie Appliquée et Environnement, LCAE-URAC18, Faculity of Science, University Mohammed Premier, Oujda 60000, Morocco; E-Mail: hammoutib@gmail.com; 6 Lab of Chemical Material, FSO, University Mohammed Premier, Oujda 60000, Morocco; E-Mail: taibi.ben.hadda@gmail.com; 7 Department of Chemistry, the University of Jordan, Amman 11942, Jordan; E-Mail: hadsal2003@yahoo.com; 8 Department of Chemistry, Faculty of Science, Benghazi University, P.O. Box 1308, Benghazi 5341, Libya; E-Mail: ahmedboshaala@yahoo.co.uk

**Keywords:** nickel(II) complex, thermal decomposition, 2,9-dimethyl-1,10-phenanthroline, nanoparticles

## Abstract

[NiCl_2_(C_14_H_12_N_2_)(H_2_O)] complex has been synthesized from nickel chloride hexahydrate (NiCl_2_·6H_2_O) and 2,9-dimethyl-1,10-phenanthroline (dmphen) as *N*,*N*-bidentate ligand. The synthesized complex was characterized by elemental analysis, infrared (IR) spectroscopy, ultraviolet-visible (UV-vis) spectroscopy and differential thermal/thermogravimetric analysis (TG/DTA). The complex was further confirmed by single crystal X-ray diffraction (XRD) as triclinic with space group P-1. The desired complex, subjected to thermal decomposition at low temperature of 400 ºC in an open atmosphere, revealed a novel and facile synthesis of pure NiO nanoparticles with uniform spherical particle; the structure of the NiO nanoparticles product was elucidated on the basis of Fourier transform infrared (FT-IR), UV-vis spectroscopy, TG/DTA, XRD, scanning electron microscopy (SEM), energy-dispersive X-ray spectrometry (EDXS) and transmission electron microscopy (TEM).

## 1. Introduction

Among transition metal oxides, nickel oxide (NiO) bulk and nano size have received considerable attention due to their wide range of applications in different fields, such as: catalysis [1–3], fuel cell electrodes and gas sensors [4–7], electrochromic films [8–10], battery cathodes [11–15] magnetic materials [16–18], and photovoltaic devices [19]. Furthermore, NiO is being studied for applications in smart windows [20], electrochemical supercapacitors [21] and dye-sensitized photocathodes [22]. Because of the quantum size and surface effects, NiO nanoparticles exhibit catalytic, optical, electronic, and magnetic properties that are significantly different than those of bulk-sized NiO particles [23–25].

1,10-Phenanthroline and its derivatives are well-known established *N*,*N*-bidentate ligands for transition metal complexation, to enrich the steric and electronic environment in such ligands different substituent function groups can be setup in their structures [22–30]. For the same reason 2,9-dimethyl-1,10-phenanthrolines and their complexes have been frequently used in the field of molecular biology and supramolecular self-assembly [22–32]. Furthermore, transition metals complexes of phenanthroline ligands are of interest to researchers because of their role in molecular scaffolding in DNA cleaving, structural studies, building blocks for the synthesis of metallo-drimers, thin films of luminescent properties, control of redox properties, analytical chemistry, and catalysis [22–32].

The starting complex and final NiO nanoparticles product before and after thermal decomposition were characterized on the basis of Fourier transform infrared (FT-IR), ultraviolet-visible (UV-vis) spectroscopy, differential thermal/thermogravimetric analysis (TG/DTA), X-ray diffraction (XRD), scanning electron microscopy (SEM), energy-dispersive X-ray spectrometry (EDXS) and transmission electron microscopy (TEM); additionally the complex was characterized by elemental analysis and X-ray single crystal structure.

In this work, we wish to report the synthesis and characterization of mononuclear *aqua*-dichloro(2,9-dimethyl-1,10-phenanthroline-κ^2^*N*,*N*)nickel(II) complex. Subsequently, direct thermal decomposition process of the desired complex precursor is one of the most important and straightforward strategies to access structurally elaborated and pure NiO nanoparticles with regular spherical particle.

## 2. Results and Discussion

### 2.1. Synthesis of the Desired Complex and NiO Nanoparticles

**Scheme 1.**
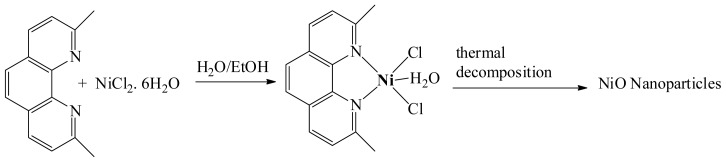
Synthesis of the complex and NiO nanoparticles.

The mononuclear 2,9-dimethyl-1,10-phenanthroline-nickel(II) complex was isolated in excellent yield by stirring equivalent amounts of 2,9-dimethyl-1,10-phenanthroline in distilled water with NiCl_2_·6H_2_O in ethanol [33,34]. NiO nanoparticles were successfully synthesized through thermal decomposition of the (2,9-dimethyl-1,10-phenanthroline)NiCl_2_ complex precursor at 400 ºC. NiO is formed via decomposition of 2,9-dimethyl-1,10-phenanthroline organic and chloride ligands in open atmosphere to NiO powder product and CO*_x_*, NO*_x_*, ClO*_x_* as expected gases bi-products. Uniform and spherical NiO nanoparticles with weak agglomeration were collected, as seen in Scheme 1.

The structures of the desired complex before and after thermal decomposition to prepare the NiO nanoparticles product were subjected to several available physical measurements.

### 2.2. Thermal Decomposition Analysis of NiCl_2_(2,9-Dimethyl-1,10-phenanthroline)·H_2_O Complex to NiO Nanoparticle

**Figure 1.**
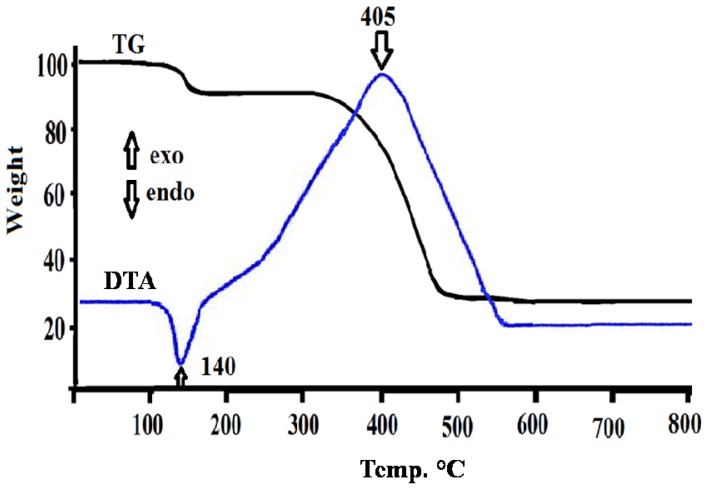
TG/DTA thermal curves of the desired complex.

The thermal properties TG/DTA of the desired complex was investigated under open atmosphere in the 0–800 ºC temperature range and heating rate of 10 ºC/min. Typical thermal TG/DTA curve is given in Figure 1.

As seen from thermal curves, no uncoordinated water molecules were detected in the complex structure in the range 50–100 ºC. One coordinated-water molecule was recorded with weight loss ~5% in the range 130–160 ºC and sharp DTA endothermic signal at ~140 ºC. Thermal decomposition study of residue complex showed no intermediate decomposition steps of the coordinated chlorides and 2,9-dimethyl-1,10-phenanthroline ligands, both ligands de-structured away from the complex to CO*_x_*, NO*_x_* and ClO*_x_* as gases by-products with one broad step decomposition at 300–500 ºC and an exothermic DTA signal at ~405 ºC; the final main product was confirmed by IR to be NiO, then subjected to several available physical measurements [16,24–27,34].

### 2.3. IR Spectral Investigation

**Figure 2.**
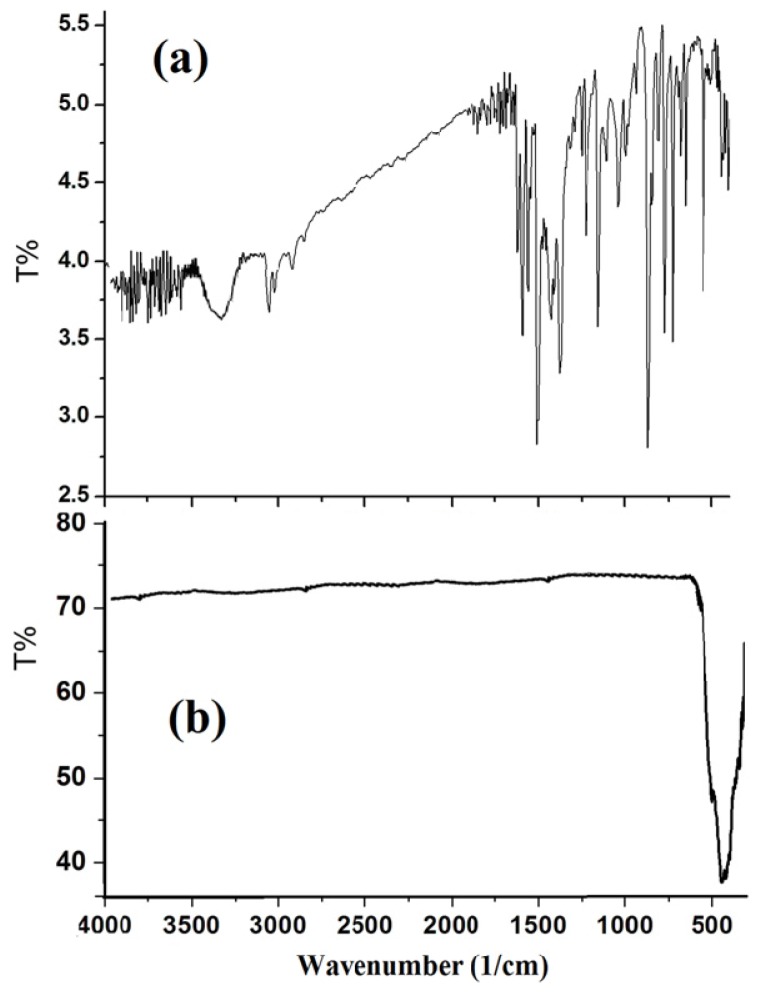
IR-KBr disk spectra (**a**) desired complex; and (**b**) NiO nanoparticles.

IR spectrum in particular showed five main sets of characteristic absorptions in the range 3410, 3090, 2940, 530 and 390 cm^−1^, which can be assigned to, coordinated-H_2_O, H–Ph, H–CH_2_, Ni–N and Ni–Cl stretching vibrations, respectively as in Figure 2a, all bands of the of (2,9-dimethyl-1,10-phenanthroline)NiCl_2_ complex disappeared after thermal decomposition at 400 ºC and a strong band at around 440 cm^−1^ is observed, as seen in Figure 2b, which was assigned to the Ni–O stretching of the octahedral NiO_6_ groups in the face center cubic NiO structure [30–33].

### 2.4. Electronic Absorption Spectral Study

**Figure 3.**
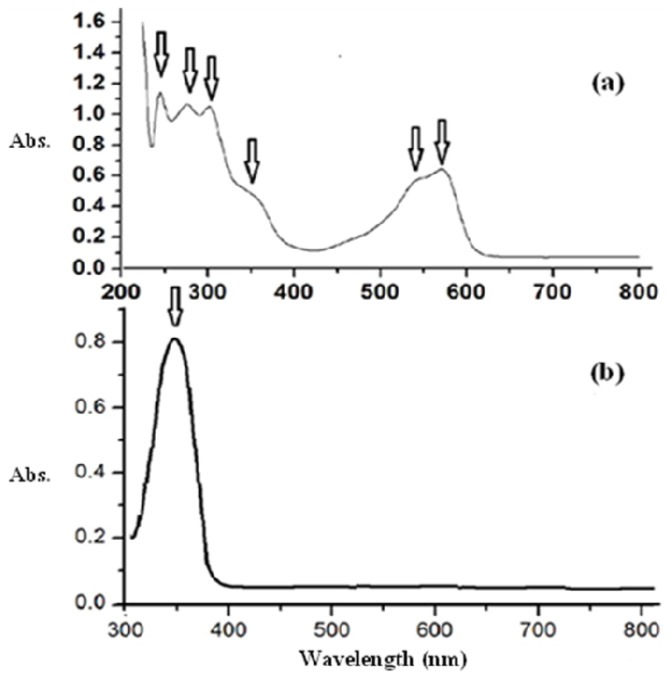
UV-vis spectrum of the desired complex (**a**) and NiO nanoparticles dissolved in water at room temperature (**b**).

The optical properties of the NiCl_2_(2,9-dimethyl-1,10-phenanthroline)·H_2_O complex was investigated by UV-vis spectroscopy (Figure 3a). For comparison, UV-vis spectra of the prepared NiCl_2_(2,9-dimethyl-1,10-phenanthroline)·H_2_O complex and NiO nanoparticles using water as solvent are also presented in Figure 3b. As expected, the aqueous solution of the starting complex in Figure 3a exhibits multiple absorptions in the UV-visible regions. The ligand displayed typical ligand-centered π→π* transitions at 240, 280 and 304 nm. Upon coordination with nickel ions, there are minor changes of these bands. The visible spectra of the desired complex was obtained at higher concentration (10^−4^ M) with the maximum absorption at 350, 530 and 560 nm can be assigned to d to d electron transition or Metal to Ligand Charge Transfer (MLCT) [32–37]. It was clearly evident that the UV-vis spectrum of the NiO nanoparticles is quite different from that starting complex, confirming the strong band that appeared at 355 nm is due to NiO nanoparticle, not Ni(II) complex. This strong absorption band is attributed to the electronic transition from the valence band to the conduction band in the NiO semiconductor [36,37]. In addition, the UV-vis spectrum of a commercial bulk NiO powder does not show any observable absorption band [36].

The optical band gap of NiO nanoparticles has been calculated from the absorption spectrum using the Tauc relation [38].

(1) $$(ɛh\nu )=\text{C}{\left(h\nu -E_{\text{g}}\right)}^{n}$$

**Figure 4.**
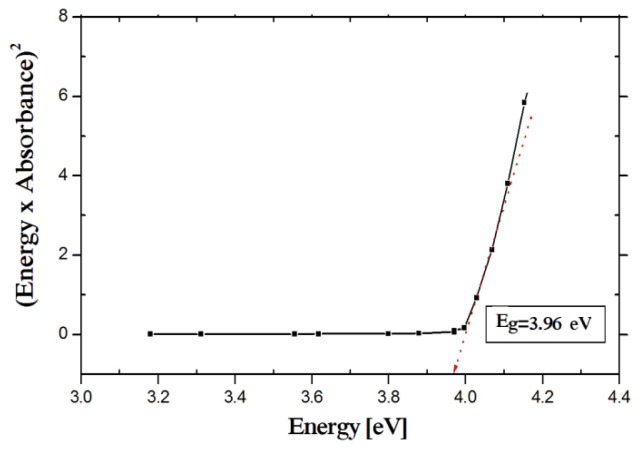
UV-vis band gap energy.

Where C is a constant, ɛ is molar extinction coefficient, *E*_g_ is the average band gap of the material and *n* depends on the type of transition. For *n* = ½, *E*_g_ in Equation (1); is direct allowed band gap. The average band gap was estimated from the intercept of linear portion of the (ɛ*h*ν)^2^*vs. h*ν plots, as shown in Figure 4.

The strongest absorption peak of the NiO prepared sample appears at around 355 nm, which is fairly blue shifted from the absorption edge of bulk NiO nanoparticle [35,36]. The band gap energy calculated from UV-absorption is 3.96 eV. This value is higher than bulk NiO *i.e.*, *E*_g_ = 3.74 eV. So it is highly agreed that the synthesized NiO is in nano scale [14,26].

### 2.5. EDX Analysis

**Figure 5.**
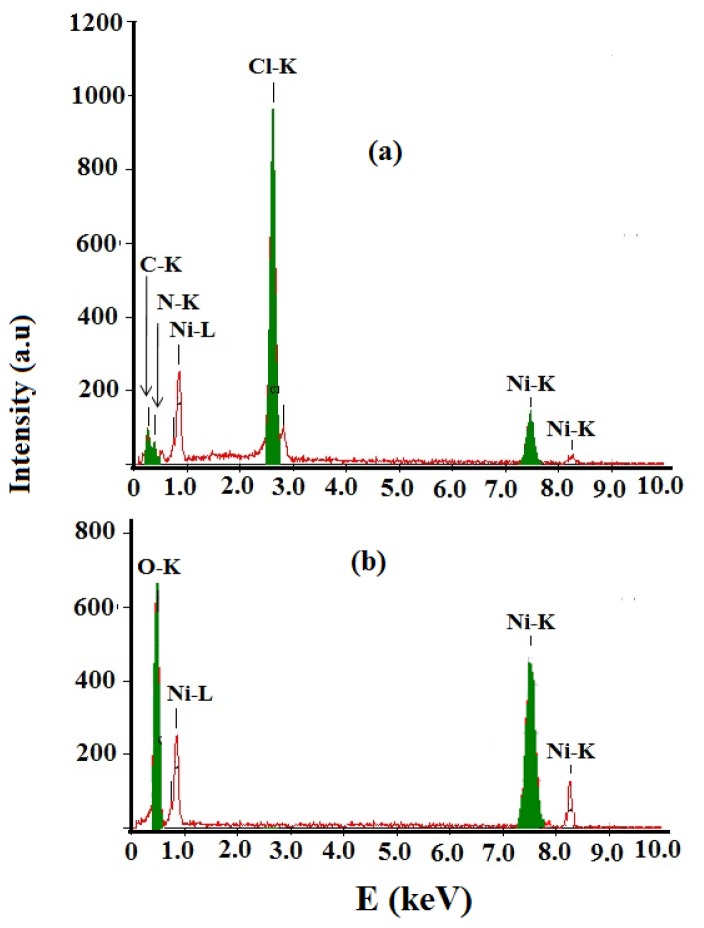
EDX spectra (**a**) NiCl_2_(2,9-dimethyl-1,10-phenanthroline)·H_2_O complex; and (**b**) NiO nanoparticles.

EDX analysis of the NiCl_2_(2,9-dimethyl-1,10-phenanthroline)·H_2_O complex and NiO nanoparticles product were represented in Figure 5. EDX of complex revealed several signals come from Ni, Cl, N, C and O, Figure 5a. After thermal decomposition process only Ni and O signals come from the NiO nanoparticles formation as seen in Figure 5b.

### 2.6. X-ray Single Crystal of NiCl_2_(2,9-Dimethyl-1,10-phenanthroline)·H_2_O Complex and XRD Powder of NiO

**Figure 6.**
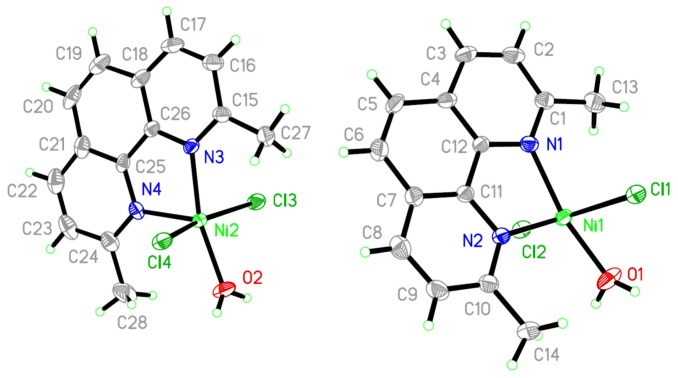
ORTEP (Oak Ridge Thermal Ellipsoid Plot) plot of two molecular units per asymmetric unit in P-1 showing atom labelling. Thermal ellipsoids are drawn at the 50% probability level.

The molecular structure is shown in Figure 6 and selected bond distances and angles are given in the Table 1. The complex was crystallized in triclinic P-1 space group.

The Ni(II) ion is five-coordinated to two N atoms of 2,9-dimethyl-1,10-phenanthroline and two Cl ions and one O atom of water. The overall geometry around each nickel center atom is in a slightly distorted triangular bipyramid configuration. Several H–O and H–Cl hydrogen-bonds were detected which may stabilized the structure of mononuclear nickel(II).

**Figure 7.**
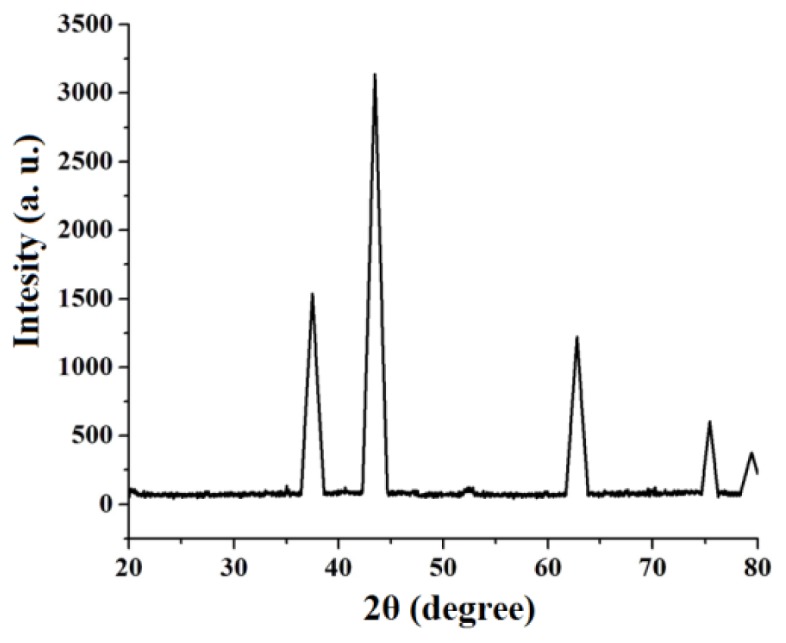
XRD patterns of NiO nanoparticles via complex thermal decomposed at 400 ºC.

Figure 7 shows powder XRD patterns of the decomposition NiCl_2_(2,9-dimethyl-1,10-phenanthroline)·H_2_O complex product at 400 ºC reveals only the diffraction peaks attributable to NiO with face-centered cubic phase at 2θ = 37.40, 43.45, 62.95, 75.40 and 79.45, Figure 7, which can be perfectly related to (111), (200), (220), (311) and (222) crystal planes, respectively (JCPDS card No. 73-1523). This finding confirms that at 400 ºC the complex was decomposed completely to nickel oxide. No peaks of impurity were found in the XRD pattern, indicating that the nanocrystalline NiO obtained via this synthesis method consists of ultrapure phase.

The average size of the NiO nanoparticles was estimated using the relative intensity peak (220) by the Debye-Scherrer equation [39], was found to be 16 nm and increase in sharpness of XRD peaks indicates that particles are in crystalline nature:

(2) D=(094λ)/(βcosθ)

Where λ is the wavelength (λ = 1.542 Å) (Cu-K_α_), β is the full width at half maximum (FWHM) of the line, and θ is the diffraction angle.

### 2.7. SEM Measurement

**Figure 8.**
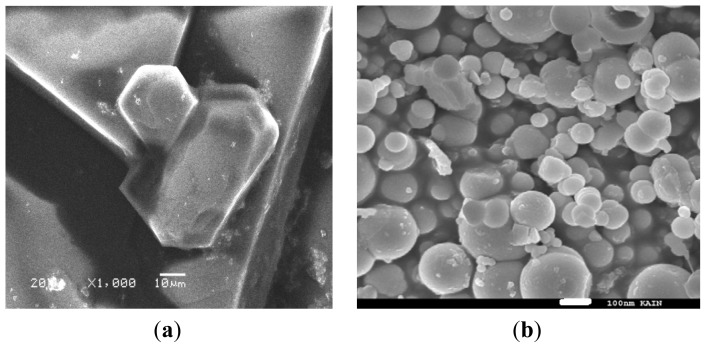
SEM micrographs (**a**) NiCl_2_(2,9-dimethyl-1,10-phenanthroline)·H_2_O complex and (**b**) NiO nanoparticles.

The SEM micrographs of the NiCl_2_(2,9-dimethyl-1,10-phenanthroline)·H_2_O complex and its decomposition product at 400 ºC are presented in Figure 8a. We observed that the starting complex powder was made of very large block crystals in different sizes. The SEM image of the product in Figure 8b clearly shows that the shape and size of particles are quite different from the precursor complex. It can be seen that the product was formed from extremely fine semi-spherical particles that were loosely aggregated. No characteristic morphology of the complex is observed, indicating the complete decomposition into the extremely fine spherical particles.

### 2.8. TEM Measurement

**Figure 9.**
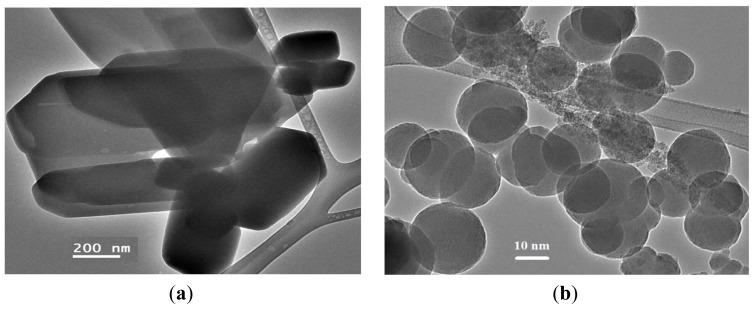
TEM micrographs (**a**) NiCl_2_(2,9-dimethyl-1,10-phenanthroline)·H_2_O complex and (**b**) NiO nanoparticles.

The TEM images of the complex and its decomposition product at 400 ºC shown on Figure 9. We observed that the TEM micrograph of the starting complex powder was made of very large block crystals in different sizes Figure 9a. Uniform NiO nanoparticles have sphere shapes with weak agglomeration Figure 9b was collected after thermal decomposition of the complex. The particle sizes possess a narrow distribution in a range from 10 to 20 nm, and the mean particle diameter is about 15 nm. Actually, the mean particle size determined by TEM is very close to the average particle size calculated by Debye-Scherer formula from the XRD pattern.

## 3. Experimental Section

### 3.1. Material and Instrumentation

2,9-Dimethyl-1,10-phenanthroline ligand and Nickel chloride hexahydrate NiCl_2_·6H_2_O were purchased from Acros, Geel, Belgium and used as received. Elemental analyses were carried out on an Elementar Vario EL analyzer (Vario EL, Donaustrass, Germany) The obtained nanoparticles were examined by a Brucker D/MAX 2500 X-ray diffractometer (Brucker, Darmstad, Germany) with Cu-K radiation (λ = 1.54 Å). The transmission electron microscopy TEM was (1001 JEOL, Maputo, Japan). The scanning electron microscopy (SEM) used a JSM-6360 ASEM (JEOL, Maputo, Japan). The IR spectra for samples were recorded using Perkin Elmer Spectrum 1000 FT-IR Spectrometer (PerkinElmer Inc., Waltham, MA, USA). Samples were measured using a TU-1901 double-beam UV-vis spectrophotometer (KFW, Haryana, India) was dispersed in water solvent.

### 3.2. General Procedure for the Preparation of the Desired Complex

A mixture of nickel chloride hexahydrate NiCl_2_·6H_2_O (Acros) (100 mg, 4.10 mmol) in distilled water (15 mL) and 2,9-dimethyl-1,10-phenanthroline (Acros) (dmphen) (80 mg, 4.20 mmol) in methanol (4 mL) was stirred for 1 h at room temperature. The solution was concentrated to about 1 mL under reduced pressure and then added to 40 mL of cooled ethanol. This causes the precipitation of (134 mg, ~92% yield) brown powder product that was filtered, and dried, and the crystals were grown by slow diffusion of ethanol into a solution of the complex in water.

### 3.3. General Procedure for the Preparation of NiO Nanoparticles

According to the TG/DTA analysis the 0.5 g of NiCl_2_(2,9-dimethyl-1,10-phenanthroline)·H_2_O complex was decomposed at 400 ºC temperatures for 0.5 h in ambient air. The decomposition products were collected for characterization.

### 3.4. Supplementary Material

Crystallographic data has been deposited with the Cambridge Crystallographic Data Centre as supplementary publication number CCDC 910435. Copies of this information may be obtained free of charge via http://www.ccdc.cam.ac.uk/conts/retrieving.html (or from the CCDC, 12 Union Road, Cambridge CB2 1EZ, UK; Fax: +44-122-3336-033; E-Mail: deposit@ccdc.cam.ac.uk).

### 3.5. X-ray Structural Analyses for the Complex

The X-ray data for complex was collected (Table 2) on Xcalibur E goniometer (Agilent Technologies, Oxford Diffraction, Oxford, UK) with enhance X-ray source and Eos CCD detector, graphite-monochromated Mo-K_α_ radiation (λ = 0.71073 Å) using five ω-scans with a total of 350 frames at temperature of 293 K. Data collection, cell parameters evaluation, data reduction and absorption were performed using CrysAlisPro, Agilent Technologies, Version 1.171.35.11 (release 16-05-2011 CrysAlis171 .NET, Oxford, UK). Structure determination was made using SHELXL programs (SHELXL-97, University of Gottingen, Gottingen, Germany) [40].

The structure was solved by direct methods and refined by full-matrix least-squares with anisotropic temperature factor, for the non-hydrogen atoms.

## 4. Conclusions

The new [NiCl_2_(C_14_H_12_N_2_)(H_2_O)] complex was subjected to thermal decomposition at low temperature of 400 ºC in an open atmosphere in order to prepare uniformed spherical NiO nanoparticles in the range of 10–20 nm. The structures of the complex and the NiO nanoparticles product were elucidated on the basis of FT-IR, UV-vis spectroscope, TG/DTA, XRD, SEM, EDX and TEM. The application of NiO nanoparticles is currently underway in our laboratory.

## Figures and Tables

**Table 1.** Selected bond distances (Å) and angles (º) of the complex.

Bond	Bond distances (Å)
Ni(II)–O(II)	2.014(2)
Ni(II)–N(III)	2.044(2)
Ni(II)–N(IV)	2.046(3)
Ni(II)–Cl(IV)	2.337(8)
Ni(II)–Cl(III)	2.347(2)
**Angles**	**Angles value (º)**
O(II)–Ni(II)–N(III)	164.61(11)
O(II)–Ni(II)–N(IV)	113.61(11)
N(III)–Ni(II)–N(IV)	81.75(10)
O(II)–Ni(II)–Cl(IV)	87.35(6)
N(III)–Ni(II)–Cl(IV)	91.91(7)
N(IV)–Ni(II)–Cl(IV)	96.63(7)
O(II)–Ni(II)–Cl(III)	87.35(6)
N(III)–Ni(II)–Cl(III)	88.27(6)
N(IV)–Ni(II)–Cl(III)	102.71(7)
Cl(IV)–Ni(II)–Cl(III)	160.48(4)

**Table 2.** Crystal data and structure refinement for the complex.

Parameters	Data
Empirical formula	C_14_H_14_C_l2_N_2_NiO
Formula weight	355.9 g/mol
Temperature	293.2(2) K
Wavelength	0.71073 Å
Crystal system	Triclinic
Space group	P-1
Unit cell dimensions	*a* = 7.5511(3) Å, α = 106.680(5)
	*b* = 11.5028(7) Å, β = 93.419(4)
	*c* = 18.9030(11) Å, γ = 103.448(5)
Volume	1,515.54(14) Å^3^
*Z* Formula units per unit cell	4
Density (calculated)	1.560 mg/m^3^
Absorption coefficient	1.628 mm^−1^
*F*(000)	728 e/cell
Crystal size	0.50 × 0.50 × 0.25 mm^3^
Theta range for data collection	2.95º to 25.02º
Index ranges	−8 ≤ *h* ≤ 8, −12 ≤ *k* ≤ 13, −22 ≤ *l* ≤ 22
Reflections collected	10,281
Independent reflections	5,327 [*R*_(int)_ = 0.0283]
Completeness to θ = 25.02º	99.8%
Absorption correction	Semi-empirical from equivalents
Max. and min. transmission	0.6864 and 0.4966
Refinement method	Full-matrix least-squares on *F*^2^
Data/restraints/parameters	5,327/8/379
Goodness-of-fit on *F*^2^	1.062
Final *R* indices [*I* > 2σ(*I*)]	*R*1 = 0.0369, *wR*2 = 0.0756
*R* indices (all data)	*R*1 = 0.0472, *wR*2 = 0.0816
Largest difference peak and hole	0.395 and −0.313 e Å^−3^
